# *In vitro* Superoxide Production by Peripheral Neutrophils from Patients with Inflammatory Bowel Disease

**DOI:** 10.1155/S0962935194000219

**Published:** 1994

**Authors:** O. H. Nielsen, D. Berild, I. Ahnfelt-Rønne

**Affiliations:** 1 Department of Medical Gastroenterology C Herlev Hospital University of Copenhagen Herlev Ringvej, Herlev DK-2730 Denmark; 2 Department of Infectious Diseases Ullevål Hospital University of Oslo Norway; 3 Department of Growth and Vascular Biology Biopharmaceuticals Division Novo Nordic Ltd Gentofte Denmark

## Abstract

Activated polymorphonuclear leucocytes, which are accumulated in inflammatory lesions of inflammatory bowel disease, produce tissue destructive, oxygen derived free radicals and other inflammatory mediators. The PMN superoxide production elicited by formyl-methionyl-leucyl-phenylalanine or the complement split product 5a were compared in IBD and healthy volunteers. Significantly reduced superoxide production was found in PMNs from patients with Crohn's disease as compared to normal controls, when fMLP or CSa were used as stimulants (p < 0.001 and p < 0.01, respectively), whereas no differences were found when ulcerative colitis patients were compared to normal controls (p > 0.05). The enhanced oxygen derived free radical production previously reported in active IBD, and especially in CD intestinal lesions, may either be due to an accumulation of productive phagocytes or to a change of the inflammatory profile of these cells when migrating into intestinal lesions, possibly due to interaction with other mediators (e.g. adhesion molecules and interleukins).


Mediators of Inflammation 3, 161–164 (1994)

### *In vitro* superoxide production by peripheral neutrophils from patients with inflammatory bowel disease

O. H. Nielsen,[1,CA] D. Berild,[2] and I. Ahnfelt-Rønne[3]

[1]Department of Medical Gastroenterology C, Herlev Hospital, University of Copenhagen, Herlev Ringvej, DK-2730 Herlev, Denmark; [2]Department of Infectious Diseases, Ullevål Hospital, University of Oslo, Norway; and [3]Department of Growth and Vascular Biology, Biopharmaceuticals Division, Novo Nordic Ltd, Gentofte, Denmark

[CA] Corresponding Author

**ACTIVATED polymorphonuclear leucocytes, which are accumulated in inflammatory lesions of inflammatory bowel disease, produce tissue destructive, oxygen derived free radicals and other inflammatory mediators. The PMN superoxide production elicited by formyl–methionyl–leucyl–phenylalanine or the complement split product 5a were compared in IBD and healthy volunteers. Significantly reduced superoxide production was found in PMNs from patients with Crohn's disease as compared to normal controls, when fMLP or C5a were used as stimulants ($p < 0.001$ and $p < 0.01$, respectively), whereas no differences were found when ulcerative colitis patients were compared to normal controls ($p > 0.05$). The enhanced oxygen derived free radical production previously reported in active IBD, and especially in CD intestinal lesions, may either be due to an accumulation of productive phagocytes or to a change of the inflammatory profile of these cells when migrating into intestinal lesions, possibly due to interaction with other mediators (e.g. adhesion molecules and interleukins).**

**Key words:** Complement 5a, Free radicals, Inflammatory bowel diseases, Neutrophils, N–formylmethionine–leucyl–phenylalanine, Receptors

### Introduction

Neutrophils and macrophages are prominent in the inflamed bowel wall of patients with ulcerative colitis (UC) and Crohn's disease (CD),[1] and it has been suggested that these phagocytes play a predominant role in the progression of tissue damage at inflammatory sites.[2] They produce a diversity of pro-inflammatory mediators, including the reactive oxygen species superoxide, hydrogen peroxide, hypochlorus acid and hydroxyl radicals.[3]

Neutrophils and macrophages migrating into the tissues originate from the circulating pool of these cells. It has been hypothesized that the cause of inflammatory bowel disease (IBD) may be related to an inherent defective function of these circulating leukocytes,[2,4] even various other factors like adhesion molecules and interleukins also may be important. It is therefore of interest to study the function of peripheral leukocytes from patients with IBD.

In the present study the capacity of circulating neutrophils from untreated UC and CD patients for superoxide production in response to challenge with two physiologically relevant stimuli, N-formylmethionine– leucyl–phenylalanine (fMLP) and complement split product 5a (C5a) was measured *in vitro*. Pathogenic roles for molecules of the bacterial cell wall products of N-formyl-methionyl peptides and of the complement cascade in IBD have earlier been proposed, but the nature and extent of their involvement in the immunopathophysiology is still undefined.[5,6]

### Materials and Methods

Human PMNs were isolated from the blood of IBD patients; 21 fulfilling the diagnostic criteria for CD,[7] 20 for UC,[8] and 29 healthy volunteers. None of the IBD patients or controls were allowed to take any drugs for the preceding 4 weeks (i.e. all IBD patients were without specific treatment with 5-aminosalicylic acid and/or glucocorticoids). Disease activity was scored according to a semiquantitative scale[9] (0 = remission, 1 = slight, 2 = moderate, and 3 = severe). The blood was drawn into EDTA (10 mM) and neutrophilic granulocytes (PMNs) were purified according to the method of Böyum.[10] Briefly, erythrocytes were allowed to sediment in methylcellulose, the buffy coat leucocytes were washed, using hypotonic lysis to remove any contaminating erythrocytes, and the mononuclear leucocytes and platelets were separated from the PMNs by gradient centrifugation in Lymphoprep (Nycomed, Oslo, Norway). More than 90% of PMNs were viable after challenge with activators (fMLP or recombinant human C5a) (Sigma Chemical Co, MO, USA), as judged from the Trypan blue exclusion test.[11]

© 1994 Rapid Communications of Oxford Ltd

Superoxide production was measured spectrophotometrically by the superoxide dismutase (SOD) inhibitable reduction of ferricytochrome c.[12] One ml aliquots of PMNs ($2 \times 10^6$ cells/ml) suspended in an NADPH assay buffer (65 mM Na/K-phosphate according to Bromberg and Pick[13] were transferred to plastic cuvettes (1 cm light path). The cells were stimulated for 5 min with either fMLP (2.3 μM) or C5a (1 μM) after previous preincubation with the priming agent cytochalasin B (100 nM, 15 min), and the production of superoxide was monitored continuously for up to 15 min at 37°C. The reduction of cytochrome C was measured by the absorbance at 550 nm, against a blank cuvette containing a similar volume of PMNs, cytochrome C, challenger and superoxide dismutase (300 IU/ml). The maximal superoxide production per min was then calculated from the rate of change of absorbance using the extinction coefficient $E_{550} = 2.1 \times 10^4$/M/cm. All experiments were performed in duplicate.

*Ethics:* The present study was performed in accordance with the Second Helsinki Declaration, and approved by the Scientific Ethical Committee of the Copenhagen County.

*Statistics:* Non-parametric statistics (medians, ranges and percentiles) were applied. Unpaired data were tested by the Mann–Whitney rank sum test and by the Kruskal–Wallis test which is a one-way analysis of variance. A significance limit of 0.05 (2α) was used.

### Results

Initial concentration–response studies showed that a maximal superoxide production was achieved with $2.3 \times 10^{-6}$ M fMLP and $10^{-6}$ M C5a, respectively. When this concentration of fMLP was used, the cells from healthy volunteers produced 9.2 (5.0–15.6), UC patients 8.2 (2.0–14.3) (N.S.) and CD patients 7.4 (3.4–12.1) ($p < 0.001$) nmol superoxide/min/$2 \times 10^{-6}$ cells (median value) (Fig. 1A). The corresponding values obtained with C5a were 7.1 (2.5–15.4), 5.3 (0.1–11.7) (N.S.) and 3.7 (0.2–9.9) ($p < 0.01$), respectively (Fig. 1B). Superoxide production was significantly impaired in the CD PMNs when comparing the three groups by a one-way variance analysis ($p < 0.03$ and $p < 0.02$ for fMLP and C5a, respectively).

When IBD patients were divided in accordance with their disease, no statistically significant correlation was found (Fig. 2).

### Discussion

Formyl peptides, of which fMLP is a major component, are the major chemotactic factor produced by *E. coli.*[14] FMLP and complement split products like C5a stimulate PMN aggregation, superoxide production, enzyme secretion and chemotaxis.[5,15–19] The PMNs have specific fMLP- and C5a receptors.[20,21] In the present study these challengers were applied within the physiologically relevant concentration range.[15,22]

The present study shows a significantly diminished superoxide production for circulating PMNs of CD patients, and a trend towards an impaired production by PMNs from UC. This appears difficult to reconcile with the hypothesis that production of reactive oxygen metabolites such as superoxide, hydroxyl and hypochlorite contribute to the tissue injury seen in active IBD.[23] The discrepancy may, however, be

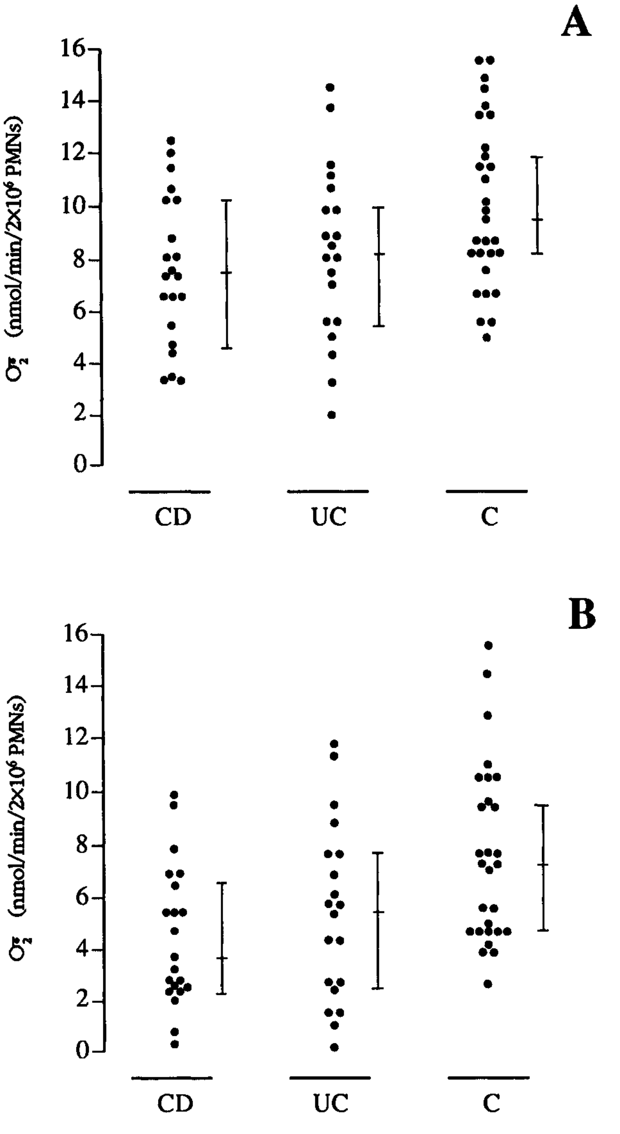

FIG. 1. Superoxide production from neutrophilic cells stimulated with either fMLP (A) or C5a (B) (nmol/min/ $2 \times 10^6$ cells) in 29 controls (C), 20 patients with ulcerative colitis (UC), or 21 patients with Crohn's disease. Medians and 25/75 percentiles are given.

explained by the high number of PMNs present at inflamed intestinal segments. Further, it is quite possible that other cell types such as monocytes/macrophages may also participate in the production of oxygen-derived free radicals (ODFR) in inflamed intestinal segments of IBD,[24] and that an interplay with other mediator systems, such as ICAM-1/LFA-1 and interleukin-8, may change the profile of peripheral PMNs when migrating into intestinal lesions. It can not be excluded that such an activation of PMNs may result in an enhanced capacity for superoxide production locally in the intestine.

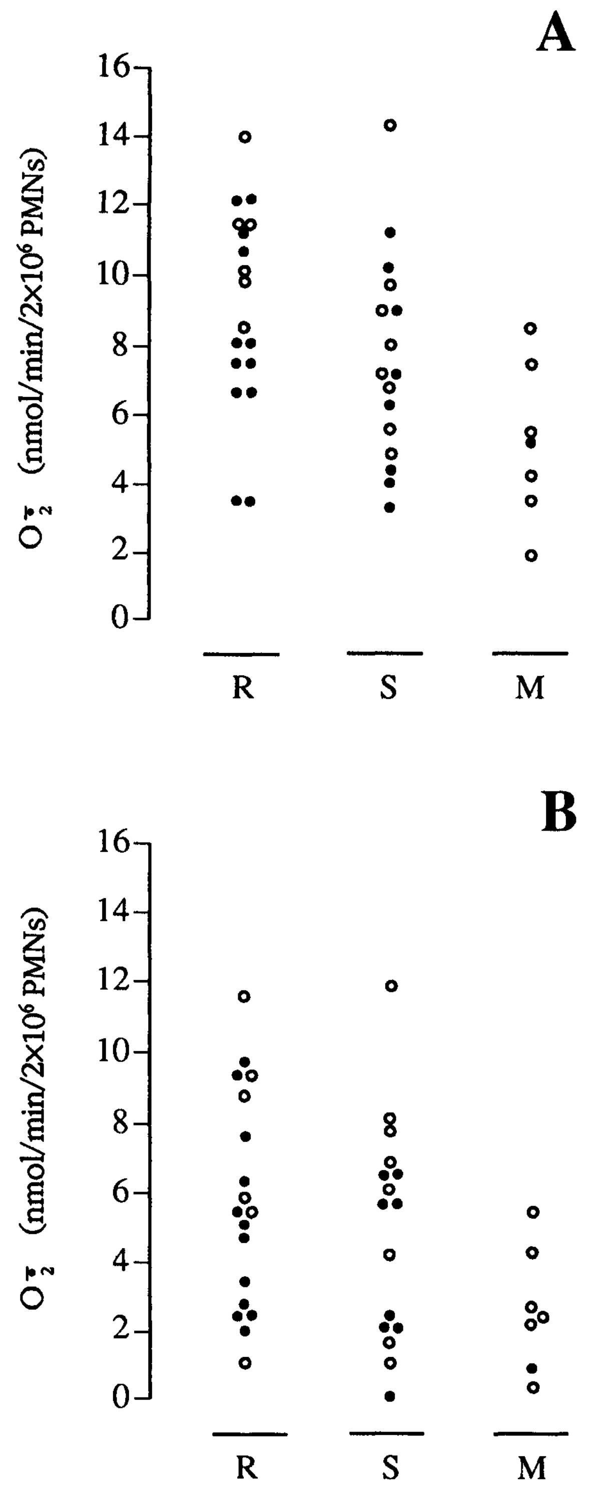

FIG. 2. Superoxide production from neutrophilic cells stimulated with either fMLP (A) or C5a (B) (nmol/min/2 x $10^6$ cells) among 18 inflammatory bowel disease patients in remission (R), 16 patients with slight (S) and 7 with moderate (M) disease activity (O = UC) (● = CD).

Earlier reports have demonstrated impaired ODFR production of PMNs from IBD patients.[25-27] Thus, using a luminol dependent chemiluminescence method, PMNs from CD and UC produced significantly lower amounts of ODFR, as compared to control patients, when stimulated with fMLP.[26]

Two other reports have shown diminished superoxide production from IBD patients stimulated with PMA, while the production of hydrogen peroxide was normal.[25,27] Further, the content of superoxide dismutase in PMNs, a cytoprotective enzyme, was also markedly diminished in both CD and UC, whereas the concentration of neutrophil elastase, a neutral protease, was normal.[25]

The present study is the first to investigate superoxide production in IBD patients who did not take any drugs. 5-aminosalicylic acid (mesalazine) and glucocorticoids may both influence PMN production of ODFR *in vivo*.[23,28]

In a Japanese study on UC, in which all patients received their normal medication, a significantly enhanced chemiluminescence was found in a subgroup with active disease.[29] However, the type II error in that study was considerable, the fMLP concentration applied was approximately 20 times higher than in the present study, and further no preincubation with cytochalasine B was performed. Methodological differences may thus explain these apparent contradictory results.

In conclusion, peripheral PMNs from CD patients have an impaired capacity to produce superoxide upon stimulation with fMLP and C5a *in vitro*. However, tissue PMNs and macrophages, which were not evaluated in the present monocellular system, may possibly be primed by other mediators of inflammation, such as leukotrienes, interleukins, adhesion molecules and lipopolysaccharides, to enhance the production of tissue destructive ODFR as seen in intestinal lesions of IBD. Further, the high numbers of phagocytes migrating into the inflamed intestinal lesions due to intestinal bacterial derived products, like fMLP and activated complement split products, may also contribute to the excess production of ODFR which is believed to be an important mediator of tissue destruction in IBD.[24,30]

### References

1. Nielsen OH. *In vitro* studies on the significance of arachidonate metabolism and other oxidative processes in the inflammatory response of human neutrophils and macrophages—with special reference to chronic inflammatory bowel disease. *Scand J Gastroenterol* 1988; **23** (suppl. 150): 1–21.
2. Schreiber S, Raedler A, Stenson WF, MacDermott P. The role of the mucosal immune system in inflammatory bowel disease. *Gastroent Clin North Am* 1992; **21**: 451–502.

3. Nielsen OH, Ahnfelt-Rønne I. Involvement of oxygen-derived free radicals in the pathogenesis of chronic inflammatory bowel disease. *Klin Wochenschr* 1991; **69**: 995–1000.
4. Harris ML, Schiller HJ, Reilly PM, Donowitz M, Grisham MB, Bulkley GB. Free radicals and other reactive oxygen metabolites in inflammatory bowel disease: cause, consequence or epiphenomenon. *Pharm Ther* 1992; **53**: 375–408.
5. Elmgreen J. Complement and function of neutrophils in chronic inflammatory bowel disease. *Dan Med Bull* 1986; **33**: 222–228.
6. Chadwick VS, Anderson RP. Inflammatory products of commensal bacteria and gastrointestinal disorders. *Dig Dis* 1990; **8**: 253–268.
7. Munkholm P. Langholz E, Nielsen OH, Kreiner S, Binder V. Incidence and prevalence of Crohn's disease in the county of Copenhagen, 1962–1987: a six fold increase in incidence. *Scand J Gastroenterol* 1992; **27** 609–614.
8. Langholz E, Munkholm P. Nielsen OH, Kreiner S, Binder V. Incidence and prevalence of ulcerative colitis in the county of Copenhagen 1962 to 1987. *Scand J Gastroenterol* 1991; **26**: 1247–1256.
9. Tvede M, Bondesen S, Nielsen OH, Rasmussen SN. Serum antibodies to *Bacteroides* species in chronic inflammatory bowel disease. *Scand J Gastroenterol* 1983; **18**: 783–789.
10. Böyum A. Isolation of leucocytes from human blood. *Scand J Clin Lab Invest* 1968; **21** (suppl. 97): 9–29.
11. DeRenzis FA, Schechtman A. Staining by neutral red and trypan blue in sequence for assaying vital and nonvital cultured cells. *Stain Technol* 1973; **48**: 135–136.
12. McCord JM, Fridovich I. Superoxide dismutase: an enzymatic function for erythrocuprein (hemocuprein). *J Biol Chem* 1969; **224**: 6049–6063.
13. Bromberg Y, Pick E. Activation of NADPH-dependent production in a cell-free system by sodium dodecyl sulfate. *J Biol Chem* 1985; **260** 13539–13545.
14. Marasco WA, Phan SH, Krutzsch H. *et al.* Purification and identification of formyl-methionyl-leucyl-phenylalanine as the major peptide neutrophil chemotactic factor produced by *Escherichia coli*. *J Biol Chem* 1984; **259**: 5430–5439.
15. Painter RG, Skjlar L, Jesaitis AJ, Schmitt M, Cochrane CG. Activation of neutrophils by N-formyl chemotactic peptides. *Fed Proc* 1984; **43**: 2737–2742.
16. O'Flaherty JT, Rossi AG, Jacobson DP, Fredman JF. Roles of $Ca^{2+}$ in human neutrophil responses to receptor agonists. *Biochem J* 1991; **278** 705–711.
17. Grinstein S, Furuya W. Receptor-mediated activation of electropermeabilized neutrophils. Evidence for a $Ca^{2+}$ and proteinkinase C-independent signaling pathway. *J Biol Chem* 1988; **263**: 1779–1783.
18. Lew PD, Monod A, Waldvogel FA, Pozzan T. Role of cytosolic free calcium and phosphilipase C in leukotriene $B_4$ stimulated secretion in human neutrophils. Comparison with the chemotactic peptide formyl-methionyl-leucyl-phenylalanine. *Eur J Biochem* 1987; **162**: 161–168.
19. Goldstein IM. Complement: biologically active products. In: Gallin JI, Goldstein IM, Snyderman R, eds. *Inflammation: Basic principles and clinical correlates, 2nd edition*. New York: Raven Press, Ltd.l 1992; 63–80.
20. Anderson T, Dahlgren C, Lew PD, Stendahl O. Cell surface expression of f-met-leu-phe receptors on human neutrophils: correlation to changes in the cytosolic free $Ca^{2+}$ level and action of phorbol myristate acetate. *J Clin Invest* 1987; **79**: 1226–1233.
21. Chenoweth DE, Hugli TE. Demonstration of specific C5a receptor on intact human polymorphonuclear leukocytes. *Proc Natl Acad Sci USA* 1978; **75**: 3943–3947.
22. Cooper NR, The complement system. In: Stites DP, Stobo JD, Wells JV, eds. *Basic and Clinical Immunology (6th edition)*. Appelton & Lange: Los Altos, CA, 1987; 114–127.
23. Ahnfelt-Rønne I. Rationales for drug development in inflammation: eicosanoids and oxygen-derived free radicals. *Dan Med Bull* 1991; **38**: 291–303.
24. Baldassano RN, Schreiber S, Johnston RB, Fu RD, Muraki T, MacDermott RP. Crohn's disease monocytes are primed for accentuated release of toxic oxygen metabolites. *Gastroenterology* 1993; **105**: 60–66.
25. Verspaget HW, Pena AS, Weterman IT, Lamers CBHW. Diminished neutrophil function in Crohn's disease and ulcerative colitis identified by decreased oxidative metabolism and low superoxide dismutase content. *Gut* 1988; **29**: 223–228.
26. Williams JG, Hughes LE, Hallett MB. Toxic oxygen metabolite production by circulating phagocytic cells in inflammatory bowel disease. *Gut* 1990; **31**: 187–193.
27. Curran FT, Allan RN, Keighley MRB. Superoxide production by Crohn's disease neutrophils. *Gut* 1991; **32**: 399–402.
28. Ahnfelt-Rønne I, Nielsen OH, Christensen A, Langholz E, Binder V, Riis P. Clinical evidence supporting the radical scavenger mechanism of 5-aminosalicylic acid. *Gastroenterology* 1990; **98**: 1162–1169.
29. Shiratora Y, Aoki S, Takada H, *et al.* Oxygen-derived free radical generating capacity of polymorphonuclear cells in patients with ulcerative colitis. *Digestion* 1989; **44**: 163–171.
30. Simmonds NJ, Rampton DS. Inflammatory bowel disease—a radical review. *Gut* 1993; **34**: 865–868.

ACKNOWLEDGEMENTS. The authors are grateful to Mrs Anni Petersen for skilful technical assistance. This study was supported by grants from direktør Jacob Madsen & hustru Olga Madsen's Foundation, handelsgartner Ove Villiam Buhl Olesen and hustru Edith Buhl Olesen's Foundation, and Else and Mogens Wedell-Wedellsborg's Foundation.

**Received 20 December 1993; accepted in revised form 19 January 1994**
